# Question Marks Left Over a Quantitative Assessment of *Apolipoprotein C3* Gene Polymorphisms

**DOI:** 10.14336/AD.2016.0502

**Published:** 2016-05-27

**Authors:** Philipp G. Sand

**Affiliations:** Department of Psychiatry, University of Regensburg, and Danuvius Klinik GmbH, Ingolstadt, Germany

Dear Editor,

On the occasion of the 2012 annual meeting of the American Society of Human Genetics in San Francisco, the scientific press concluded to a sobering "genetic influences on disease remain hidden" after discussing, among others, the progress made in the field of cardiovascular genetics [1]. In a recent attempt to resolve some of the inconsistent findings, Zhang and coworkers [2] have presented a quantitative analysis of *APOC3* variants in coronary heart disease. Unfortunately, only a fraction of the previously published data have been considered and, using the authors’ inclusion criteria, over 10000 alleles are missing from the investigation [3-8]. What is more, incorrect allele counts have led to biased effects for the *Sst*I polymorphism, causing the risk-enhancing allele to become protective [9] and *vice versa* [10]. Allele counts for the T-455C variant also differ from the published data [11] and are further compromised by duplicates from overlapping samples [11,12]. With regard to both T-455C and C-482T, the vast majority of allele frequencies reported in Table 1 of the article [2] are either in error [11,13-18], missing [12], or entirely fictional [19]. Finally, failure to identify C3175G as a synonym of the *Sst*I polymorphism has led to the omission of more alleles from a publication which served to extract data on T-455C and C-482T [13].

On the whole, the article calls for numerous issues to be ironed out prior to claiming, or to refuting, significant effects of the three *APOC3* variants on coronary heart disease susceptibility.
