# Supplementary material for: Question Marks Left Over a Quantitative Assessment of Apolipoprotein C3 Gene Polymorphisms
Source: Aging Dis. 2016 May 27;7(3):216–9. doi: 10.14336/AD.2016.0502 (PMC4898917; doi:10.14336/AD.2016.0502)
Supplement: Related texts

# Correction: Association Between Apolipoprotein C-III Gene Polymorphisms and the Risk of Coronary Heart Disease: a Meta-analysis

**Published:** 2016-05-27

**Authors:** Jing-Zhan Zhang, Xiang Xie, Yi-Tong Ma, Ying-Ying Zheng, Yi-Ning Yang, Xiao-Mei Li, Zhen-Yan Fu, Chuan-Fang Dai, Ming-Ming Zhang, Guo-Ting Yin, Fen Liu, Bang-Dang Chen, Min-Tao Gai

**Affiliations:** Department of Cardiology, First Affiliated Hospital of Xinjiang Medical University, Urumqi, 830054, China

Aging Dis. 2016 Jan 2;7(1):36-44.

doi: 10.14336/AD.2015.0709.

In light of Dr. Sands’ letter, we have reanalyzed data from our meta-analysis of the association between apolipoprotein C-III gene polymorphisms and coronary heart disease (CHD)[1]. Our original database searches did not recover several references cited by Dr. Sand[2-5], which we have now included, and we were unable to extract data useful for our analysis from two other references [6-7]. We have reexamined additional studies referred to by Dr. Sand [8-14] and recalculated our results accordingly.

The new calculations still show an association between the APOC3 SstI polymorphism and CHD under allelic contrast (*P* <0.0001, OR = 1.17, 95% CI = 1.08-1.27), dominant genetic (*P* = 0.001, OR = 1.17, 95% CI = 1.07-1.29), and recessive genetic (*P* = 0.01, OR = 1.36, 95% CI = 1.07-1.73) models; between the APOC3 T-455C polymorphism and CHD under allelic contrast (C vs. T, *P* = 0.007, OR = 1.19, 95% CI = 1.05-1.35) and dominant genetic (CT+CC vs. TT, *P* = 0.0004, OR = 1.26, 95% CI = 1.11-1.43), but no longer recessive genetic (CC vs. CT+TT, *P* = 0.12, OR = 1.25, 95% CI = 0.95-1.66) models; and no association between the APOC3 C-482T polymorphism and CHD under allelic contrast (T vs. C, *P* = 0.59, OR =1.02, 95%CI = 0.95-1.10), dominant genetic (TT+TC vs. CC, *P* = 0.17, OR = 1.08, 95% CI = 0.97-1.20)or recessive genetic (TT vs. TC+CC, *P* = 0.41, OR = 0.94, 95% CI = 0.82-1.09) models. Our meta-analysis suggests that the APOC3 SstI polymorphism significantly increases, the APOC3 T-455C polymorphism may increase, and the APOC3 C-482Tpolymorphism shows no association with CHD susceptibility.

We sincerely apologize for the errors in our article.

PMID: 26816662 [PubMed] PMCID: PMC4723232

## References

[1] ZhangJZ, XieX, MaYT, ZhengYY, YangYN, LiXM, FuZY, DaiCF, ZhangMM, YinGT, LiuF, ChenBD, GaiMT (2016). Association between Apolipoprotein C-III Gene Polymorphisms and Coronary Heart Disease: A Meta-analysis. Aging Dis, 7:36-44.2681666210.14336/AD.2015.0709PMC4723232

[2] MiettinenHE, KorpelaK, HämäläinenL, KontulaK (1994). Polymorphisms of the apolipoprotein and angiotensin converting enzyme genes in young North Karelian patients with coronary heart disease. Hum Genet, 94:189-192.791391110.1007/BF00202868

[3] BaroniMG, BerniA, RomeoS, ArcaM, TesorioT, SorropagoG, Di MarioU, GaltonDJ (2003). Genetic study of common variants at the Apo E, Apo AI, Apo CIII, Apo B, lipoprotein lipase (LPL) and hepatic lipase (LIPC) genes and coronary artery disease (CAD): variation in LIPC gene associates with clinical outcomes in patients with established CAD. BMC Med Genet, 4:8.1296494310.1186/1471-2350-4-8PMC201027

[4] BhanushaliAA, DasBR (2010). Influence of genetic variants in the apolipoprotein A5 and C3 gene on lipids, lipoproteins, and its association with coronary artery disease in Indians. J Community Genet, 1:139-148.2246024610.1007/s12687-010-0025-xPMC3185992

[5] AshokKumarM, SubhashiniNG, SaiBabuR, RameshA, CherianKM, EmmanuelC (2010). Genetic variants on apolipoprotein gene cluster influence triglycerides with a risk of coronary artery disease among Indians. Mol Biol Rep, 37:521-527.1970169310.1007/s11033-009-9728-7

[6] MasanaL, FebrerG, CavannaJ, BaroniMG, MarzW, HoffmannMM, ShineB, GaltonDJ (2001). Common genetic variants that relate to disorders of lipid transport in Spanish subjects with premature coronary artery disease. Clin Sci, 100:183-190.11171287

[7] WongWM, HaweE, LiLK, MillerGJ, NicaudV, PennacchioLA, HumphriesSE, TalmudPJ (2003). Apolipoprotein AIV gene variant S347 is associated with increased risk of coronary heart disease and lower plasma apolipoprotein AIV levels. Circ Res, 92:969-975.1267681610.1161/01.RES.0000069688.94567.7A

[8] OlivieriO, StranieriC, BassiA, ZaiaB, GirelliD, PizzoloF, TrabettiE, ChengS, GrowMA, PignattiPF, CorrocherR (2002). ApoC-III gene polymorphisms and risk of coronary artery disease. J Lipid Res, 43:1450-1457.1223517610.1194/jlr.m200145-jlr200

[9] LiXD, SunZH, YangZY, et al (2006). APOAl/C3 gene cluster polymorphisms and risk of coronary artery disease. Chin J Gerontol, 26:745-747.

[10] HanTL (2011). A5/C3 Gene cluser and the correlation to the patients with coronary artery disease in ShanDong coastal areas. Shandong University, 1-50.

[11] MartinelliN, TrabettiE, BassiA, GirelliD, FrisoS, PizzoloF, SandriM, MalerbaG, PignattiPF, CorrocherR, OlivieriO (2007). The -1131 T>C and S19W APOA5 gene polymorphisms are associated with high levels of triglycerides and apolipoprotein C-III, but not with coronary artery disease: an angiographic study. Atherosclerosis, 191:409-417.1668204110.1016/j.atherosclerosis.2006.04.009

[12] LiuHK, LiFX, ZhangSZ, et al (2005). Association of Sst I Polymorphism in Apolipoprotein C3 Gene withHypertriglyceridaemia in Coronary Atherosclerotic Heart Disease and Type II Diabetes Mellitus in ChinesePopulation. Yi Chuan Xue Bao, 32:11-18.15715433

[13] LiuHK (2005). Study on the association of single nucleotide polymorphisms in apolipoproteinAPOAl/C3/A4/A5 gene cluster with coronary heart disease and type II diabetes. Sichuan University, 1-116

[14] TobinMD, BraundPS, BurtonPR, ThompsonJR, SteedsR, ChannerK, ChengS, LindpaintnerK, SamaniNJ (2004). Genotypes and haplotypes predisposing to myocardial infarction: a multilocus case-control study. Eur Heart J, 25:459-467.1503912510.1016/j.ehj.2003.11.014
